# The impacts of probiotics in eradication therapy of *Helicobacter pylori*

**DOI:** 10.1007/s00203-022-03314-w

**Published:** 2022-11-07

**Authors:** Xiaofen Bai, Minjie Zhu, Yajun He, Tengyan Wang, Da Tian, Jianchang Shu

**Affiliations:** 1 Department of Gastroenterology, Guangzhou Red Cross Hospital, Jinan University, Guangzhou, China; 2 Department of Clinic Laboratory, Guangzhou Red Cross Hospital, Jinan University, Guangzhou, China; 3 Department of Gastroenterology, Guangzhou Panyu Central Hospital, Guangzhou, China

**Keywords:** Probiotics, Eradication therapy, Helicobacter pylori

## Abstract

Helicobacter pylori (*H. pylori*) is a well-known pathogen that infects approximately half of the world’s population. It is a pathogenic agent with potential health hazards related to diverse diseases, especially digestive diseases, such as chronic gastritis, peptic ulcer, and gastric carcinoma. In clinical, antibiotics are commonly applied in eradication therapy of *H. pylori*. However, the increase in antibiotic resistance and side effects has induced the failure of eradication therapy. Recent studies have shown that probiotic supplementation has promising application prospects. It can restore the gastrointestinal microbiota balance and prevent dysbacteriosis caused by antibiotics. Furthermore, it has been reported to have direct or indirect inhibitory effects on *H. pylori*. Probiotics may have a beneficial effect on *H. pylori* eradication. However, the strain, dosages, duration times, and safety of probiotic supplementation need further study before clinical applications.

## Introduction

*H. pylori* is a bacterium that infects approximately half of the world’s population (Hooi et al. 2017). Although most *H. pylori*-positive individuals remain asymptomatic, it is known that *H. pylori* is related to the development of various clinical conditions, such as peptic ulcers, gastric adenocarcinomas, and mucosa-associated lymphoid tissue lymphomas (Brito et al. 2019; Ford et al. 2020). It was first explicitly formulated in the Kyoto Global Consensus Report that *H. pylori* gastritis should be considered an infectious disease, whether the affected individual had any symptoms, complications, or consequent illnesses (Sugano et al. 2015). Following the European Maastricht V/Florence consensus statement, anyone with *H. pylori* infection should undergo the eradication treatment (Malfertheiner et al. 2017). Since the 1997 Maastricht consensus, standard triple therapy has been employed in most countries as the first-line regimen of *H. pylori* eradication. The quadruple therapy was exercised later by adding bismuth, which was also used as the first-line regimen (Malfertheiner et al. 1997). However, the increase in antibiotic resistance and the reduction of compliance with therapeutic regimens have induced eradication therapy failure and changes in the therapeutic regimen. Therefore, alternative treatments have been proposed to eradicate *H. pylori*, including novel antibiotics or classical ones in different combinations, which have been used in regular clinical practice as novel and more effective treatments.

Probiotics are considered as living microorganisms. Adequate amounts of probiotics are beneficial to health (Hill et al. 2014). Studies on human and animal models have revealed the mechanisms of probiotics, including the production of antibacterial substances, competitive inhibition of adherence to intestinal epithelium of pathogens and toxins, immunological regulation, maintenance of intestinal epithelial homeostasis, etc. (Yan and Polk 2020; Quigley 2019). Besides, studies have shown that probiotics could suppress drug-resistant bacteria growth and drug-resistant gene transmission (Kunishima et al. 2019). Meanwhile, fecal microbial transplantation decolonized some pathogenic antibiotic-resistant organisms (Wieers et al. 2019; Millan et al. 2016). Many clinical trials have reported that using specific probiotics alone could diminish the *H. pylori* bacterial load. The specific probiotics supplementation in the *H. pylori* standard eradication treatment protocol may improve the eradication rate and reduce the side effects caused by antibiotics (Homan and Orel 2015). A meta-analysis of 19 randomized controlled trials found that adjunctive therapy of multiple strain probiotics could improve eradication rates of *H. pylori* and prevent the occurrence of adverse events and antibiotic-associated diarrhea, but not all the mixtures were effective (McFarland et al. 2016).

This review was performed to summarize recent studies on the status of *H. pylori* infection, diagnosis, therapeutic regimen, the effects of probiotics on *H. pylori*, mechanism, and application recommendations. Unlike the systematic review which has focused on one aspect to analysis, multiple aspects of *H. pylori* were mentioned and a number of literatures, which include reviews, guidelines, meta-analyses, cell and animal studies, and clinical trials, were cited in our review.

## Status, Pathogenesis, and treatment of *H. pylori* infection

### Status of H. pylori infection

*H. pylori* is a gram-negative bacterium that infected approximately 4.4 billion individuals worldwide in 2015, based on regional prevalence estimates (Hooi et al. 2017). Reports of infection prevalence rates range widely among geographic regions, reaching the highest levels in developing countries and showing a well-established relationship with socioeconomic status and hygiene conditions (Roberts et al. 2016; Alzahrani et al. 2014). *H. pylori* infection prevalence in the United States was estimated to be around 35% by Hooi et al. while the infection rate was higher in Africa, Central America, Central Asia, and Eastern Europe (Hooi et al. 2017). A cross-sectional study conducted in the United Arab Emirates found a prevalence of 41% in healthy children and adults (Melese et al. 2019). The most significant studies performed in Korea involved 24,471 subjects and had a seroprevalence of 41.5% (Lim et al. 2018). A 12-year retrospective study in a tertiary center study from East China on 3252 subjects showed a prevalence of 27.5% (Tang et al. 2019). In Malaysia, the *H. pylori* infection rate had a specific ethnic structure—*H. pylori* prevalence was the highest among Indians (> 50%), followed by Chinese (40–50%), whereas Malays had a relatively low prevalence of infection, approximately 10–20%(Goh 2018). Conversely, Bolivia had up to 80%, despite the inclusion of children, suggesting an early acquisition in childhood in the country. A study from Brazil reported that the prevalence rates of the infection reached 70–90% in adults and children aged 5–10 years old (Coelho et al. 2018; Sivapalasingam et al. 2014). Although reports have shown a continuous decline in *H. pylori* prevalence in many regions worldwide, including Korea, China, Iran, and Austria (Leja et al. 2019), management of this infection remains a formidable challenge due to antibiotic-resistant increases.

### Pathogenesis of H. pylori infection

#### Virulence factors

*H. pylori* can activate the immune response, which would induce gastritis, peptic ulcers, and gastric cancer. It may also contribute to immune thrombocytopenic purpura and increase the risk of acute coronary syndrome, cerebrovascular disease, and neurodegenerative disease (Tsay and Hsu 2018). However, various virulence factors and virulence genes of *H. pylori* are involved in the pathological process of gastrointestinal disorders caused by *H. pylori* (Brito et al. 2019).

Urease is a critical factor that enables bacterial colonization in the gastric mucosa, protects the bacterium from gastric acidity, promotes bacterial nutrition, and generates the proton motive force during the hydrolysis of urea (Ansari and Yamaoka 2019). Adding to urease's impact on acid neutralization, its catalysis has pathogenicity. Ammonia impairs cell junctions, breaches cellular integrity, and damages gastric epithelium. CO2 protects the bacterium from the bactericidal activity of metabolic products like nitric oxide and intracellular killing by phagocytes (Ansari and Yamaoka 2019; Debowski et al. 2017).

The *cag* pathogenicity island contains genes encoding a secreted effector protein CagA and components of the type IV secretion system Cag T4SS (Cover et al. 2020). CagA injects the cell via the pilus formed by T4SS and induces cellular alterations (Ansari and Yamaoka 2020). Meanwhile, CagA can facilitate carcinogenesis by modulating apoptosis, disrupting cell polarity, and promoting genetic instability (Sterbenc et al. 2019). Moreover, CagA is highly associated with the induction and further progression of EMT in gastric mucosa, which is related to greater invasiveness properties. However, consensus recommends considering CagA as an effector protein rather than a toxin because CagA could not act without the bacterial cell, elicit acute damage to host cells, and counteract the activities of the established *H. pylori* toxin VacA (Knorr et al. 2019).

VacA is a cytotoxin involved in pore formation, and its gene expression can be observed in all *H. pylori* strains, but toxin activity differed depending on exposure time to the host cells. VacA promotes autophagy pathways in cells in acute exposure. However, VacA improves impaired autophagosome appearance and induces intracellular vacuole formation, which enables *H. pylori* survival in the host cells (Baj et al. 2020). *H. pylori* has been proven to downregulate autophagic protein expression and inhibit autophagy, while the VacA toxin plays a vital role in it (Ricci 2016). Except for autophagy, VacA is involved in many processes of damaging gastric epithelial cells, such as alterations in mitochondrial functioning, apoptosis, and necrosis (Foegeding et al. 2016). Abdullah et al*.* revealed that a synergistic effect of VacA and CagA on accumulating CagA in VacA induced impaired autophagosomes (Abdullah et al. 2019).

DupA is a *H. pylori* virulence factor with multifunctional biological activities, and it can be considered an essential biomarker in duodenal ulcer (DU) (Alam et al. 2020). The prevalence of the DupA gene in strains was higher in patients with duodenal ulcers than in patients with gastritis or gastric cancer (Ansari and Yamaoka 2017). However, some studies have shown that DupA is a protective factor that prevents gastric carcinogenesis (GC) and the proliferation and growth of GC cells by over-activating the mitochondria-mediated apoptotic pathway and the high tolerance of DupA-positive strains to the acidic gastric microenvironment (Talebi et al. 2012; Queiroz et al. 2011).

In addition, *H. pylori* protease, PqqE, could disrupt the structure and function of tight junctions of epithelium and damage gastric epithelial integrity by cleaving junctional adhesion molecule A (Marques et al. 2021). The expression of outer membrane proteins (OMPs) helps *H. pylori* attach to gastric epithelial cells in the primary stage and increases *H. pylori* virulence (Xu et al. 2020). Encoded by the *hopH* gene, OipA is associated with bacterial adherence, colonization, induction, and the progression of gastrointestinal disorders. Further, it may regulate CagA and VacA syntheses (Al-Maleki et al. 2017).

### Immunological aspects

*H.pylori* infection always results in intense and complex host immune responses, but it hardly induces clearance of the infection. *H. pylori* is thought to downregulate inflammation and control the host's immune response via various virulence factors involved in provoking and maintaining a pro-inflammatory immune response (Kusters et al. 2006). *H. pylori* infection could activate the innate immunity. Various *H. pylori* antigens and products, such as lipoteichoic acid, lipoproteins, lipopolysaccharide, HSP-60, NapA, DNA, and RNA, bind to gastric cell receptors located on epithelial cell membranes. *H. pylori* LPS is a primary activator of the innate immune response in epithelial cells, and it could activate NF-κB via TLR2 and TLR4-mediated recognition (Smith 2014; Nagashima et al. 2015).

Many pathogenic effects of *H. pylori* infection are related to chronic active inflammation, which is controlled and maintained by complex interplay of pro-inflammatory and anti-inflammatory mediators. In *H. pylori*-positive patients, studies found a Th1-polarized response, characterized by lack of IL-4 and growth of gamma interferon, tumor necrosis factor, IL-1β, IL-6, IL-7, IL-8, and IL-10 (Brito et al. 2019). IL-1β is a potent pro-inflammatory cytokine and the most potent inhibitor of acid secretion up to now. It induces gastric secretion reduction, which is associated with corpus-predominant colonization by *H. pylori*, causing gastritis, atrophic gastritis, and even gastric cancer (El-Omar et al. 2000).

### Treatment of H. pylori infection

Most commonly, antibiotic therapy is recommended for eradication therapy. Triple therapy, which is based on combining two antibiotics plus one proton pump inhibitor (PPI) with a duration of 7–14 days, remains the standard treatment protocol (Malfertheiner et al. 2017; Talebi 2017). However, the increase in antibiotic application worldwide has induced antibiotic resistance among the bacterium, including *H. pylori*, inducing a reduction in the success rate of first-line anti-*H. pylori* therapies(Siddique et al. 2018). A study showed that, in most WHO regions, the pooled prevalence of both primary and secondary resistance of *H. pylori* to clarithromycin, metronidazole, and levofloxacin is > 15%—this is the common threshold for choosing alternative empiric regimens (Savoldi et al. 2018). It was reported that *H. pylori* resistance rate to metronidazole in China increased by approximately 50% between 2000 and 2014, and clarithromycin resistance increased from 14.8% in 2000 to 52.6% in 2014 (Thung et al. 2016). The eradication rate of traditional triple therapy based on metronidazole and clarithromycin was less than 80% (Chey et al. 2017). A network meta-analysis on the first-line treatment of *H. pylori* infection showed that the eradication rate of standard triple therapy with clarithromycin and amoxicillin or metronidazole for seven days was only 73% rather than the required quality criterion of 80% (Li et al. 2015). Therefore, addressing antibiotic resistance has become a major factor in *H. pylori* eradication success.

A systematic review and meta-analysis of 45 randomized controlled trials (RCTs), including a total of 7722 patients, provided evidence that 14 days was the optimum duration for clarithromycin-containing triple therapy (PPI, clarithromycin and amoxicillin or metronidazole/tinidazole) (Farup et al. 2002). The pooled eradication rate of the 14-day regimen significantly exceeded that of the 7-day regimen (81.9% vs. 72.9%). All the international guidelines agree that clarithromycin-containing triple therapy remains useful in the first-line treatment of *H. pylori* infection, with extending to 14 days (Malfertheiner et al. 2017; Chey et al. 2017; Fallone et al. 2016). Bismuth quadruple therapy and concomitant therapy are the best first-line empirical treatments in areas with high clarithromycin resistance and individuals with previous application of macrolides, or the 14-day clarithromycin-containing triple therapy is also a proper regimen (Zagari et al. 2021). However, a study comparing the standard 7-day triple therapy and the 14-day triple therapy with the 7-day regimen with bismuth revealed that the eradication rate of 14-day triple therapy (89%) exceeded that of 7-day standard triple therapy (79%). Nevertheless, 14-day triple therapy did not achieve the targeted 90% eradication rate in the intention-to-treat (ITT) analysis. The supplement of bismuth in the 7-day standard triple therapy did not improve the eradication rate(Leow et al. 2018). The eradication rates of different regimens varied in subpopulations with resistant infections (Graham and Dore 2016).

The eradication rate of different regimens (14-day triple therapies, sequential therapies, concomitant therapies, bismuth quadruple therapies, levofloxacin triple therapies, or 7-day vonoprazan triple therapies) exceeds 95% in subpopulations with susceptible infections (Hu et al. 2017a). More so, the *H. pylori* infection eradication rate with first-line treatment decreased below 80% in many countries, even less than 70% in some regions (Hu et al. 2017b). Recently, some new treatment regimens have been proposed, such as triplet therapy with vonoprazan, five-combination therapy, high-dose dual therapy, and standard triple therapy with probiotics (Hu et al. 2017b). More studies are needed to prove the safety and effectiveness of these emerging treatments.

## Roles of probiotics in *H. pylori* eradication therapy

### Effects on eradication rate and side effects

Recently, many studies have shown that probiotic supplementation could improve the eradication rate and reduce the side effects caused by antibiotics. The changes in the microbiome induced by antibiotics could induce diarrhea and other side effects, which could be avoided by probiotic supplementation and prevent antibiotic-related adverse events (Ianiro et al. 2014, 2016). Moreover, compared with placebos, probiotics have the therapeutic effect of *H. pylori* eradication (Goderska et al. 2018). It was reported that taking probiotics alone can diminish bacterial load, whereas applying probiotics with antibiotics can improve the eradication rate and alleviate side effects (Homan and Orel 2015; Song et al. 2019). However, other studies found that probiotic or sulforaphane with triple therapy for *H. pylori* infection neither increased the eradication rate nor decreased the incidence of adverse events (Chang et al. 2020; Mukai et al. 2020).

### Probiotic monotherapy

Zhang et al*.* performed a study that enrolled 150 subjects infected with *H. pylori* to assess the efficacy and safety of monotherapy in eradication treatment with *Clostridium butyricum*, *Bacillus coagulans,* or *C. butyricum* plus *B. coagulans* for eight weeks. ITT analysis revealed that the three groups achieved similar eradication rates, comparable compliance rates, and adverse events during treatment (Zhang et al. 2020). Lee et al*.* also suggested no significant inhibitive effects of the three probiotic strains (*L. acidophilus*, *L. rhamnosus*, and *L. sporogenes*) on *H. pylori* (Lee et al. 2017). However, following the research by Yoon's team, reduction in *H. pylori* density and histologic inflammation improvement could be observed in patients treated with fermented milk containing *L. paracasei* HP7 and *G. glabra* (Yoon et al. 2019). In a randomized double-blind placebo-controlled clinical trial, the decrease in the mean HpSA and HpSA titer exhibited a significant difference between *Saccharomyces boulardii* and the control group, thereby indicating that *Saccharomyces boulardii* could positively reduce the colonization of *H. pylori* in the human gastrointestinal system, but it is incapable of eradication as monotherapy (Namkin et al. 2016). A meta-analysis reported that taking probiotics alone could reduce the bacterial load and eradicate *H. pylori* with a 14% eradication rate, which was far from satisfactory clinically (Losurdo et al. 2018). Therefore, probiotic monotherapy cannot be used in *H. pylori* eradication therapy, though it could inhibit bacterium growth.

### Probiotics with PPI

An open-label single-center study performed on *L. reuteri* plus pantoprazole twice a day for eight weeks cured 13.6% of patients with *H. pylori* infection by ITT analysis and 14.2% by per-protocol (PP) analysis. The overall urease activity assessed before and after 4–6 weeks of therapy showed a significant reduction with a mean of 38.8 vs. 25.4 by a one-tailed test (*P* = 0.002) (Dore et al. 2014). Another similar controlled study found that *L. reuteri* DSMZ 17,648 plus pantoprazole for eight weeks achieved a similar efficacy as standard triple therapy for 14 days. The symptoms of satiety, bloating, pain, and anxiety score changes in the two groups had no statistical significance. *L. reuteri* may be a good alternative to antibiotics, and it can be used to eradicate *H. pylori* infection in patients with chronic dyspepsia (Muresan et al. 2019). However, Dore et al*.* studied that the cure rate of taking PPI with *Lactobacillus reuterii* instead of antibiotics or bismuth was 12% (Dore et al. 2019a). Two studies evaluating whether the treatment of PPI plus probiotics rather than antibiotics would eradicate *H. pylori* infections were halted because the cure rates were far below the acceptable. Besides, it was also found that the therapy of PPI plus probiotics could not provide a clinically meaningful rate of *H. pylori* eradication (Opekun et al. 2018; Dore et al. 2019b).

### Probiotics with standard eradication treatment

Many studies have attempted to elucidate probiotics' role as supplements in *H. pylori* eradication therapy, but the results are various. In a Japanese retrospective study, 468 patients with *H. pylori* infection were divided into three groups, PPI/amoxicillin (AMX)/clarithromycin (CLR), vonoprazan (VPZ)/AMX/CLR, and PPI/AMX/CLR/probiotics, respectively. The study showed a significant difference in the eradication rate between the PPI plus probiotics group and the PPI group in ITT analysis. The main side effects were diarrhea, eruption, and stomatitis, and no significant differences were observed between the three groups (Mukai et al. 2020). A study from China using traditional triple therapy plus probiotics or placebo among patients with *H. pylori*-positive peptic ulcers shared a similar conclusion that triple therapy with probiotics in treating *H. pylori*-positive peptic ulcer could significantly improve the *H. pylori* eradication rate with less adverse reaction (Ma et al. 2015). A study performed by Goran Hauser et al*.* drew a similar conclusion (Hauser et al. 2015). Probiotics with sequential treatment could also enhance the eradication rate and reduce side effects, such as diarrhea (Cekin et al. 2017; Seddik et al. 2019). The cure rates of the quadruple therapy containing bismuth and the treatment of Gastrus^®^ plus two antibiotics and PPI were 79.6% and 88% by ITT analysis. By PP analysis, the rates were 84.8% and 95.7%, respectively. Moreover, the patient’s compliance was exceptionally nice, and side effects were mild in both regimens (Dore et al. 2019b). Bismuth-containing quadruple therapy (BCQT) with probiotic supplementation could achieve excellent eradication rates and produce fewer side effects (Zhu et al. 2018, 2017), even in patients who experienced failing treatment before (Liu et al. 2020). A meta-analysis showed that, compared with the control group (only BCQT), the probiotic group (BCQT with probiotic supplementation) had a higher eradication rate with statistical significance (*P* = 0.000), and fewer adverse reactions were reported in the probiotic group than in the control group (*P* = 0.000) (Si et al. 2017).

Regarding the efficacy of standard eradication treatment with probiotics, the results remain contradictory. In a study from Spain, 209 patients were prescribed eradication therapy (10-day triple or non-bismuth quadruple concomitant therapy) and randomly received probiotics (*Lactobacillus plantarum* and *Pediococcus acidilactici*) or placebo. The eradication rates were similar in the two groups (placebo 95% vs. probiotic 97%), and no differences were observed in compliance or side effects (McNicholl et al. 2018). Among the patients with *H. pylori*-positive chronic gastritis or peptic ulcer but without clarithromycin resistance, the eradication rates and the frequencies of adverse events were similar in the triple therapy group and the triple therapy with probiotics group by ITT analysis and PP analysis (Chang et al. 2020). McNicholl et al*.* also revealed no significant differences were observed between the control and probiotic groups regarding efficacy and side effects (McNicholl et al. 2018). Some studies have shown that triple therapy with probiotics only improved the cure rate but had no impact on reducing side effects (Grgov et al. 2016; Francavilla et al. 2014; Efrati et al. 2012). However, other studies showed the reverse in reducing side effect incidence but not improving the eradication rate (Zhang et al. 2015; Chotivitayatarakorn et al. 2017).

A meta-analysis that included 45 RCTs and 6997 participants reported that the overall eradication rates of the control and probiotic groups were 72.08% and 82.31%, thus reviewing that standard therapy application with probiotics was associated with the increased eradication rate by per-protocol set analysis or intention-to-treat analysis. Besides, adverse event incidence was 36.27% in the control group and 21.44% in the probiotic group. The specific reduction in adverse events ranged from 30 to 59% and was statistically significant (Zhang et al. 2015). The results of clinical studies and meta-analyses had a discrepancy. Several studies have shown that probiotic supplementation during anti-*H. pylori* treatment effectively improved *H. pylori* eradication rate and minimized the incidence of therapy-related adverse events related to specific strains of the bacterium (McFarland et al. 2016; Lu et al. 2016a; Konorev et al. 2016; Feng et al. 2017; Shi et al. 2019). Lau et al. reported that probiotic supplementation significantly increased the eradication rate by 12.2%. They decreased the risk of diarrhea, nausea, vomiting, and epigastric pain, but no significant differences were observed in efficacy between the various types of probiotics (Lau et al. 2016). However, in a study by Lu et al. compared with the placebo group, probiotics combined with standard therapy did improve the adverse effects of diarrhea and nausea but did not increase the *H. pylori* eradication rate (Lu et al. 2016b).

### The application of probiotics in children with H. pylori infection

Some studies have applied probiotics as supplementation in *H. pylori* eradication therapy in children. Studies have shown that probiotics with triple therapy for *H. pylori* eradication in children could significantly enhance the *H. pylori* eradication rate and reduce side effect incidence (Ahmad et al. 2013; Wang and Huang 2014). Tolone et al. reported there were significant differences in the side effects of the control and probiotic groups, while no statistical differences were found in the eradication rate in both groups (Tolone et al. 2012). However, in a meta-analysis that included five studies, 484 pediatric patients concluded that *Lactobacillus* supplementation with standard triple therapy could increase the *H. pylori* eradication rate and reduce the incidence of therapy-related diarrhea in children (Fang et al. 2019). Probiotics supplementation in triple therapy for children with *H. pylori*-positive may have beneficial effects on eradication treatment and therapy-related side effects, particularly diarrhea (Li et al. 2014).

### Effects on gut microbiota

The human gut microbiota theory showed gut microbes could modulate human physiological activities in dynamic balance, such as nutrient absorption, energy metabolism, immune function, and neurological landscapes (Adak and Khan 2019). *H. pylori* infection changes the microflora of gastric and intestinal (He et al. 2016; Shin et al. 2020), and eradication therapy induces gut microbiota disorders (Olekhnovich et al. 1902; Ma et al. 2021), but probiotic supplementation may protect and recover the gut microbiota (Ji and Yang 2020). Recently, animal tests and clinical trials have shown that probiotics positively affect the host's gut microbiota by maintaining the gastric microbiota balance during *H. pylori* infection and treatment (Zhu and Liu 2017; Chen et al. 2019). Liou JM et al*.* showed that α-diversity reduced significantly and β-diversity markedly altered at the end of triple therapy, concomitant therapy, and bismuth quadruple therapy, compared with baseline (Liou et al. 2019). Wu et al*.* found that the diversity of gut microbiota decreased remarkably in patients with *H. pylori* who only underwent triple therapy. Concurrently, probiotics supplementation of *Bacillus subtilis* and *E. faecalis* inhibited the reduction (Wu et al. 2019). According to Oh et al*.*, the proportional change of functional gene families in the control group exceeded that in the probiotic group after *H. pylori* eradication treatment. The functional alterations of gut microbiota may be linked to the reduction in intestinal irritation and maintenance of bacterial diversity (Oh et al. 2016). Wang et al*.* found that prominent quantitative and qualitative alterations in the gut microbiota were observed after eradication treatment, whether standard therapy or probiotic supplementation, but most of the changes reverted on day 71 (Wang et al. 2017). Although a meta-analysis reported that microbial diversity decreased in the short-term follow-up, no research data could confirm subsequent alterations (Ye et al. 2020).

## Mechanisms of probiotics in *H. pylori* eradication therapy

Probiotics have been proven effective and beneficial for several gastrointestinal diseases, including *H. pylori* infection, diarrhea caused by antibiotics, or *clostridium difficile* and *pouchitis*, while the precise mechanism requires exploration (Sebastian 2017). Many vitro and vivo experiments have shown that various probiotics have bacteriostatic and bactericidal activity against *H. pylori* through bacterial cells or metabolites (Chen et al. 2019; Asgari et al. 2020, 2018; Saracino et al. 2020; Urrutia-Baca et al. 2018). The antagonism between probiotics and *H. pylori* is achieved via immunological and non-immunological regulation mechanisms, including production of antimicrobial substances, mucosal barrier, and adhesion competition (Goderska et al. 2018; Qureshi et al. 2019; Eslami et al. 2019).

### Immunological mechanism

*H. pylori* infection-induced inflammatory diseases were associated with the sustained expression of inflammatory factors, which had no effect on *H. pylori* eradication but continued inflammatory response (Brito et al. 2019). Probiotics modified the immunological response by modulating anti-inflammatory cytokine secretion, thereby inducing inflammation activity reduction (Wiese et al. 2012; Garcia-Castillo et al. 2018). The IL-8 release was the initial manifestation of the cytokine response, which induced the migration of neutrophils and monocytes to the mucosa. Numerous studies have found that probiotic strains, such as *L. acidophilus*, *L. bulgaricus*, *L. gasseri,* and *L. rhamnosus*, could strengthen the expression of the anti-inflammatory cytokine IL-10 (Zhao et al. 2018) and reduce the expression of IL-8 in *H. pylori*-infected cells via modulating the TLR4/IκBα/NF-κB pathway (Song et al. 2019; Chen et al. 2019; Yarmohammadi et al. 2021; Whiteside et al. 2021). A study showed that lactic acid-producing bacteria (LAB) treatments reduced the *H. pylori* loads, *vacA* gene expression, *H. pylori* specific IgA, and IgM levels in the stomach, alongside the serum levels of IFN-γ and IL-1β. The multi-LAB treatment recovered and increased the levels of some serum fatty acids and amino acids, which were important in immune functions modulation (Lin et al. 2020). Animal experiments revealed that *Lactobacillus* significantly reduced IL-6 levels but increased the IL-10 level and repaired mucosal damage (Zhou et al. 2021; Park et al. 2020). Moreover, 16S rRNA gene sequencing revealed that *H. pylori* relative abundance could be significantly decreased by *L. plantarum* ZJ316 administration (Zhou et al. 2021). Therefore, probiotics have a preventive and mitigating effect on the inflammation caused by *H. pylori* infection.

### Non-immunological mechanism

#### Antimicrobial substances

Antimicrobial production is an essential function of probiotics. Some probiotic strains could produce various antimicrobial compounds, such as short-chain fatty acids (SCFAs), hydrogen peroxide, nitric oxide, and bacteriocins, which would inhibit *H. pylori* growth via antibacterial substances (Homan and Orel 2015). During carbohydrate metabolism, probiotics could produce short-chain fatty acids, such as acetic, propionic, and lactic acids, which would lower gastric pH (Zhou et al. 2021). Organic acid could inhibit *H. pylor* growth and suppress urease activity (Rezaee et al. 2019). Furthermore, catalase induced oxygen radical production, which interfered with *H. pylori* enzyme activity and produced oxidative damage in *H. pylori*-infected cells (Ji and Yang 2020; Song et al. 2018). Moreover, bacteriocins production is the primary factor in *H. pylori* inhibition by probiotics. Boyanova et al*.* found that *Lactobacillus* produced heat-stable bacteriocin-like inhibitory substances (BLISs), which inhibited the development of antibiotic-sensitive and antibiotic-resistant strains (Boyanova et al. 2017). Reuterin compounded by *L. reuteri* inhibited the growth of *H. pylori* and downregulated the expression of the virulence genes *vacA* and flaA (Urrutia-Baca et al. 2018).

#### Mucosal barrier

The mucins and large complex glycoproteins defend the gastrointestinal mucosa epithelium against toxic substances and pathogens. Probiotics protect the mucosal barrier from damage by modifying the expression of mucus and epithelial junction proteins, and releasing bioactive molecules to stabilize the barrier (Qureshi et al. 2019). IgA produced by probiotic strains could help strengthen the mucosal barrier against pathogen invasion. Vitro studies showed probiotics could upregulate tight-junction proteins and promote mucous secretion to stabilize the mucous layer by increasing the expressions of muc1, muc2, muc3, and muc5 (Goderska et al. 2018; Hanisch et al. 2014; Suez et al. 2019; Zhang et al. 2014; Dhar and McAuley 2019). Therefore, probiotics could restore the mucosal permeability of gastric mucosa and prevent *H. pylori* colonization.

#### Competition for adhesion

Adhesion to the host tissue was a vital process of *H. pylori* colonization of the gastric mucosa. Studies have shown that probiotics, such as LAB, *Lactobacillus,* and *Streptococcus thermophilus,* reduced adhesion to *H. pylori* epithelial cells (Chen et al. 2019; Rezaee et al. 2019; Marcial et al. 2017). Probiotics hinder *H. pylori* from binding to epithelial cells in different ways, such as antimicrobial substance secretion, competition of adhesion sites, or nutrients(Qureshi et al. 2019). Takeda et al*.* found the level of the CagA virulence protein of *H. pylori* in MKN45 cells, and some viable *H. pylori* adhering to MKN45 cells decreased with *Lactobacillus paracasei* 06TCa19 supplementation(Takeda et al. 2017). Moreover, the 06TCa19 strain notably increased some lactic acids in the supernatant of MKN45 cells. Lactic acid, which was released from the 06TCa19 strain, inhibited the adhesion of *H. pylori* to MKN45 cells, and prevented the *H. pylori* CagA from inserting into the cells. *Saccharomyces boulardii* contained selective neuraminidase activity, which could remove the α(2–3)-linked sialic acid and suppress the *H. pylori* adherence to duodenal epithelial cells, because sialic acid could hold the ligands of *H. pylori* adhesion (Sakarya and Gunay 2014).

## Guidelines for the clinical use of probiotics

Many systematic reviews and meta-analyses indicated that standard *H. pylori* eradication therapy with probiotic supplementation was adequate for the growth of eradication rates and prevention of adverse effects. The present meta-analyses are inconsistent because different eradication regimens were applied in the included random control trials, and diverse probiotics were used, making it impossible to assess strain specificity. Recent guidelines revealed that probiotic supplementation was evaluated to manage *H. pylori* infection (Malfertheiner et al. 2017; Fallone et al. 2016; Zagari et al. 2015). In the Italian guideline for managing *H. pylori* infection, “Some probiotics reduce adverse effects during *H. pylori* eradication therapy” is stated (Evidence level 3a; Grade of recommendation B) (Zagari et al. 2015). The Tronto Consensus recommends routinely adding probiotics to eradication therapy to reduce adverse events or increasing eradication rates in patients with *H. pylori* infection (Fallone et al. 2016). While in Maastricht V/Florence Consensus Report, only certain probiotics could effectively minimize gastrointestinal side effects caused by *H. pylori* eradication therapies and may advantageously affect *H. pylori* eradication. The specific strains should be chosen only upon the basis of a revealed clinical efficacy (Malfertheiner et al. 2017).

## Outlook

Because of the high prevalence and severe hazard of *H. pylori* infection, it is necessary to eradicate *H. pylori*. However, the effect of anti-*H. pylori* therapy is far from satisfactory. Probiotics supplementation with standard eradication therapy has been proven to improve cure rates, decrease side effects, and maintain the host gut microflora balance, but the results are contradictory in clinical trials and meta-analyses for the diversity of antibiotic regimens, probiotic strains, host susceptibility, and other factors. Therefore, to improve the *H. pylori* eradication effect, more relevant experiments are needed to explore the optimal selection and appropriate dosage of probiotics and antibiotics.

## Data Availability

Not available.

## Availability of data and materials

Not available.
